# The Mitochondria-to-Cytosol H_2_O_2_ Gradient Is Caused by Peroxiredoxin-Dependent Cytosolic Scavenging

**DOI:** 10.3390/antiox10050731

**Published:** 2021-05-06

**Authors:** Laura de Cubas, Valeriy V. Pak, Vsevolod V. Belousov, José Ayté, Elena Hidalgo

**Affiliations:** 1 Oxidative Stress and Cell Cycle Group, Universitat Pompeu Fabra, C/ Dr. Aiguader 88, 08003 Barcelona, Spain; laura.decubas@upf.edu (L.d.C.); jose.ayte@upf.edu (J.A.); 2 Laboratory of Experimental Oncology, Pirogov Russian National Research Medical University, 117997 Moscow, Russia; valeriy.pak@embl.de (V.V.P.); belousov@fccps.ru (V.V.B.); 3 Department of Metabolism and Redox Biology, Shemyakin-Ovchinnikov Institute of Bioorganic Chemistry, 117997 Moscow, Russia; 4 Federal Center for Brain Research and Neurotechnologies, 117997 Moscow, Russia; 5 Institute for Cardiovascular Physiology, Georg August University Göttingen, 37073 Göttingen, Germany

**Keywords:** H_2_O_2_ gradients, genetically encoded fluorescent H_2_O_2_ reporter, HyPer7, roGFP-Tpx1.C169S, mitochondria

## Abstract

Fluorescent protein-based reporters used to measure intracellular H_2_O_2_ were developed to overcome the limitations of small permeable dyes. The two major families of genetically encoded redox reporters are the reduction-oxidation sensitive green fluorescent protein (roGFP)-based proteins fused to peroxiredoxins and HyPer and derivatives. We have used the most sensitive probes of each family, roGFP2-Tpx1.C169S and HyPer7, to monitor steady-state and fluctuating levels of peroxides in fission yeast. While both are able to monitor the nanomolar fluctuations of intracellular H_2_O_2_, the former is two-five times more sensitive than HyPer7, and roGFP2-Tpx1.C169S is partially oxidized in the cytosol of wild-type cells while HyPer7 is fully reduced. We have successfully expressed HyPer7 in the mitochondrial matrix, and it is ~40% oxidized, suggesting higher steady-state levels of peroxides, in the low micromolar range, than in the cytosol. Cytosolic HyPer7 can detect negligible H_2_O_2_ in the cytosol from mitochondrial origin unless the main H_2_O_2_ scavenger, the cytosolic peroxiredoxin Tpx1, is absent, while mitochondrial HyPer7 is oxidized to the same extent in wild-type and *∆tpx1* cells. We conclude that there is a bidirectional flux of H_2_O_2_ across the matrix and the cytosol, but Tpx1 rapidly and efficiently scavenges mitochondrial-generated peroxides and stops their steady-state cytosolic levels rising.

## 1. Introduction

Hydrogen peroxide (H_2_O_2_) has been traditionally linked to toxicity, but it also participates in signaling events by both regulating different physiological processes and activating antioxidant cascades (for reviews, see [1,2,3,4]). Generation and scavenging of H_2_O_2_ have been a matter of study for many decades, as well as the fluxes of peroxides between different cellular compartments.

Mitochondria is an important source of the production of reactive oxygen species. Both electron transport chain (ETC) components and other mitochondrial enzymes, such as 2-oxoglutarate dehydrogenase, pyruvate dehydrogenase, and the mitochondrial membrane forms of glycerol 3-phosphate dehydrogenase, contribute to the formation, directly or indirectly, of H_2_O_2_ (for a review, see [5]). When derived from the ETC and from other sources, peroxides are formed after spontaneous or superoxide dismutase-catalyzed dismutation from superoxide radicals. Another important source of H_2_O_2_ is the family of NADPH oxidases, normally referred to as NOX enzymes. Finally, a wide range of non-mitochondrial proteins, such as cytochrome P450 enzymes or xanthine oxidase, and organelles different from mitochondria, such as the endoplasmic reticulum, can all produce H_2_O_2_ (for a review, see [6]). 

The quantitative contribution of these organelles and proteins to the final steady-state levels of H_2_O_2_ is unknown. Traditionally the ETC has been considered the main producer of reactive oxygen species, with 5% of the electron accidentally leaking to oxygen from transiently reduced ETC intermediates [7], while other predictions suggest a 0.15% electron leakage [8]. While the analysis of Mn-SOD knockout mice experiments strongly suggested that mitochondrial ROS generation is a physiologically significant process in vivo [9,10], the group of Imlay has proposed, using *Escherichia coli*, that the contribution of the ETC to H_2_O_2_ production is not major [11]. 

Most of the studies estimating mitochondrial H_2_O_2_ production have been performed in vitro with isolated mitochondria and with the aid of ETC inhibitors. Based on their use, the topology of ROS production has been predicted. It has been demonstrated that inhibition of complex I produces superoxide towards the matrix [12]. Regarding complex III (CIII), it was first proposed that the addition of antimycin A or other inhibitors caused superoxide generation towards the matrix [13]. However, the crystal structure of CIII revealed that the semiquinone, reducing oxygen to superoxide at complex III [14], faces the IMS [15], and indeed ROS are produced at both sides of the inner membrane upon treatment with CIII inhibitors [8,16,17,18]. The specific ETC sites causing mitochondrial ROS production in vivo in the absence of ETC inhibitors are unclear. 

Regarding H_2_O_2_ scavenging, cells have developed efficient systems to metabolize peroxides. They are mainly based, although not exclusively [19], on two types of chemistries: heme-based catalases and thiol-based peroxidases (peroxiredoxins and glutathione peroxidases) [20]. While catalases depend on the heme group to promote peroxide scavenging, peroxidases use their highly reactive thiol groups, which have to be recycled at the expense of the reduced cofactor. The amount, relevance, and localization in different cellular compartments vary depending on the cell type. In fission yeast, we have established that the cytosolic peroxiredoxin Tpx1 is the major H_2_O_2_ scavenger during physiological growing conditions, and growth of cells lacking Tpx1 on solid plates is halted under aerobic conditions [21]. Tpx1 is a highly abundant typical 2-cysteine peroxiredoxin [22,23], which together with catalase constitutes the main line of defense against H_2_O_2_ [24]. Using biochemical and mathematical approaches [25], as well as the ultrasensitive H_2_O_2_ reporter roGFP2-Tpx1.C169S [26], we have determined that the cytosolic concentrations of peroxides that arrest the growth of cells lacking Tpx1 are between 0.1 and 0.3 µM. Catalase is the next barrier of detoxification when the steady-state levels of peroxides are increased in *∆tpx1* cells [24] or when toxic extracellular peroxides are applied to wild-type cultures [26]. The only glutathione peroxidase encoded by the *Schizosaccharomyces pombe* genome has a minor secondary role when high doses of extracellular peroxides are applied [24]. 

The final steady-state levels of peroxides will not only depend on synthesis and scavenging but also on subcellular localization. Biological membranes have limited permeability to H_2_O_2_, and compartment-specific scavenging activities further enhance the gradients across membranes. We have established that the fission yeast cytoplasmic membrane creates a permeability gradient of extracellular-to-intracellular peroxides of 40:1, but scavenging by the peroxiredoxin Tpx1, the main H_2_O_2_ detoxifier in fission yeast, enhances the gradient up to 300:1 [25]. 

Different concentrations of peroxides, in a space and time context, will drive physiological events, redox signaling, or toxicity. Measurements of intracellular H_2_O_2_ based on the use of permeable fluorescent dyes are controversial [27]. More than a decade ago, several groups decided to investigate the use of protein-based reporters to measure intracellular redox potentials and, later on, H_2_O_2_ levels [28]. The two major families of genetically encoded redox reporters are: the reduction-oxidation sensitive green fluorescent protein (roGFP)-based proteins [28] and HyPer and derivatives, based on the *Escherichia coli* H_2_O_2_ sensor OxyR [29]. Both families of probes suffer ratiometric fluorescence changes, which can be easily monitored with fluorescent microscopy, flow cytometry, or fluorescence plate readers. The specificity and sensitivity of roGFP to sense oxidants has been improved in the last years by fusing real H_2_O_2_ protein sensors, carrying mutations to limit their reduction rates, such as Orp1 or the peroxiredoxins Tsa2∆Cr or Tpx1.C169S [26,30,31]; fluorescence changes of roGFP fused to H_2_O_2_ sensors or of HyPer are specific for H_2_O_2_, and other oxidants such as superoxide, oxidized glutathione or other reactive oxygen species are not able to induce them. Regarding HyPer and derivatives, a new family member has been recently reported, HyPer7, which is based on *Neisseria meningitidis* OxyR [32]; as for the original HyPer, HyPer7 has been tested in mammalian cells, and the sensitivity of this biosensor is much higher than the original HyPer. 

We have expressed the most sensitive biosensors of each family, roGFP2-Tpx1.C169S [26] and HyPer7 [32], in the same model system, *S. pombe*. Both probes are exquisitely sensitive in sensing moderate fluctuations of H_2_O_2_ induced by environmental interventions, although the former, roGFP2-Tpx1.C169S, is ~5-times more sensitive than HyPer7. This superb sensitivity allows roGFP2-Tpx1.C169S to clearly sense the leakage of H_2_O_2_ from the mitochondria to the cytosol upon the addition of the electron transport chain inhibitor antimycin A. Thanks to the use of our biosensors, we propose that the concentration of peroxides in the cytosol of wild-type cells is ~3–6 nM, while the steady-state H_2_O_2_ levels within the mitochondrial matrix are ~0.1–0.15 µM. Importantly, the extent of oxidation of HyPer7 is equilibrated up to ~60% in the matrix and cytosol of cells lacking Tpx1, indicating that scavenging by the peroxiredoxin Tpx1 causes the matrix-to-cytosol peroxide gradient.

## 2. Materials and Methods

### 2.1. Fission Yeast Growth Media and Genetic Manipulations

For all the experiments, cells were grown in filtered minimal medium (MM) at 30 °C as described previously [33], supplemented with cysteine in auxotrophic strains. The strains used in this study and their genotypes are provided in Table S1.

### 2.2. Generation of Plasmids Used in This Study

The plasmids described here express fluorescent reporters under the control of the constitutive *sty1* promoter [24], and are episomal with an average of 7–8 plasmid copies per cell [34]. According to previous quantifications of other proteins expressed from the same promoter [25], the intracellular protein concentration arising from these constructs is in the order of 2–10 µM. Plasmids p605 and p407.C169S, allowing the expression of HyPer and roGFP2-Tpx1.C169S, respectively, have been previously described [26]. We cloned a synthetic open reading frame (supplied by IDT Technologies, Coralville, IA, USA) coding for HyPer7 [32] downstream of the *sty1* promoter and an HA-coding sequence, yielding plasmid p728 (allowing the expression of HA-HyPer7). We then inserted a PCR-amplified DNA fragment from *S. pombe* cDNA encoding the mitochondrial targeting signal of Aco1, its 60 N-terminal amino acids, ahead of the HA-coding sequence, yielding plasmid p730 (allowing the expression of MTS-HA-HyPer7). To express catalase in the mitochondrial matrix, we purified an insert from p419 [24], including *sty1* promoter-driven *ctt1*, and cloned it in the *ura4* episomal vector pREP.42x, inserting after that the coding sequence for MTS from Aco1, yielding p790.

### 2.3. Growth of Strains Expressing HyPer or roGFP Derivatives for Fluorescence Determination

For wild-type backgrounds, standard MM-based early stationary phase pre-cultures were diluted in filtered MM to reach an OD_600_ of 1 after 4–5 duplications. For strain *Δtrx1*, auxotrophic for cysteine, MM aerobic cultures contained 38 µg/mL of cysteine. The fluorescence of 190 µL of these cultures, at an OD_600_ of 1, was directly monitored in 96-well plates in a FLUOstar OMEGA (BMG Labtech, Ortenberg, Germany) as described below. 

### 2.4. In Vivo Measurement of Basal and Induced Oxidation of HyPer and roGFP-Derivatives

**Figure 1:**
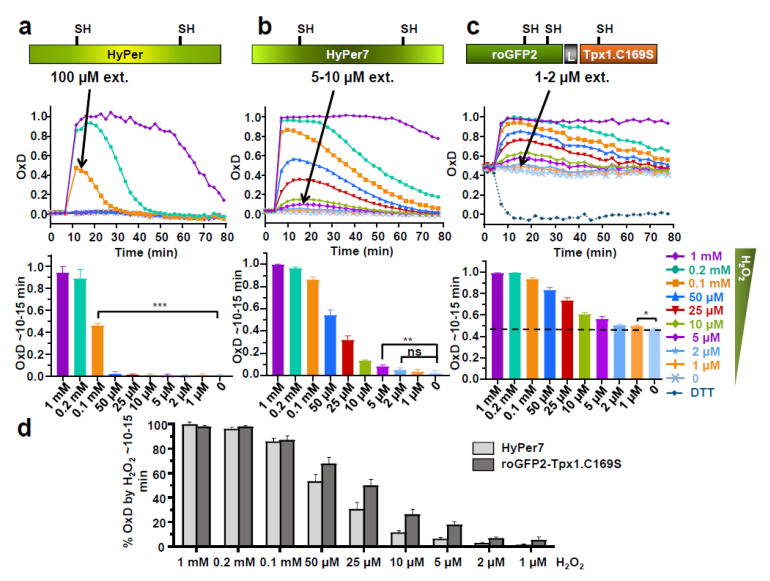
Expression of HyPer (**a**), HyPer7 (**b**) and roGFP2-Tpx1.C169S (**c**) in fission yeast. Wild-type strain HM123. was transformed with plasmids p605, p728, and p407.C169S to express HyPer, HyPer7, and roGFP2-Tpx1.C169S, respectively. The indicated extracellular concentrations of H_2_O_2_ were directly added to MM cultures at an OD_600_ of 1 in 96-well imaging plates, and growth proceeded at 30 °C for the time indicated with shaking. The degree of probe oxidation (amount of probe oxidized per 1) is indicated in the Y-axis (OxD). The minimum concentrations of H_2_O_2_ capable of causing probe oxidation are indicated with arrows. For each strain, average data from three biological replicates are shown, with error bars (S.D.) displayed in Figure S1. Graph bars (bottom panels of **a**–**c**) represent the average OxD between 10 and 15 min for each treatment. Statistical significance was calculated between the indicated samples with an unpaired Student’s *t*-test with P-values of 0.05 (*), 0.01 (**), and 0.001 (***); ns, non-significant. (**d**) Percentage of oxidation of HyPer7 and roGFP2-Tpx1.C169S upon H_2_O_2_ treatments. Data from panels b and c are represented as the average of the percentage of oxidation between 10 and 15 min for each treatment, as described in Materials and Methods.

**Figure 2:**
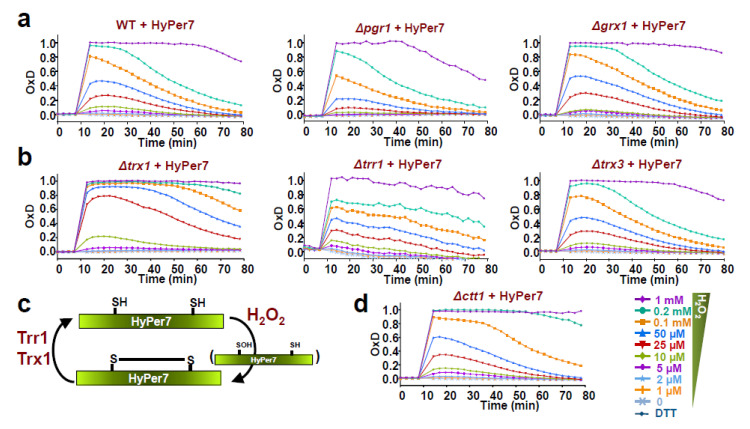
HyPer7 is recycled by the thioredoxin system. (**a,b**) Strains HM123 (WT), AD88 (∆*pgr1*), SG154. (∆*trx1*), SB469 (∆*grx1*), SG152 (∆*trr1*), and MC184 (∆*trx3*), transformed with plasmid p728 expressing cytosolic HyPer7, were treated or not with H_2_O_2_ and oxidation of the reporter was estimated as described in Figure 1. (**c**) Scheme depicting the role of the thioredoxin system as the electron donor in HyPer7 recycling. (**d**) Strain EP160 (∆*ctt1*) transformed with plasmid p728 expressing cytosolic HyPer7 was treated or not with H_2_O_2_, and oxidation of the reporter was estimated as described in Figure 1. For each strain, average data from three biological replicates are shown, with error bars (S.D.) displayed in Figure S2.

**Figure 3:**
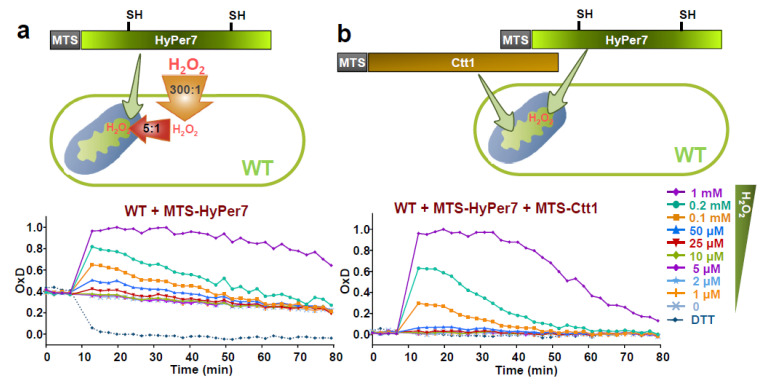
Mitochondrial MTS-HyPer7 is partially oxidized in basal conditions. (**a**) Strain HM123 (WT) transformed with plasmid p730 expressing MTS-HyPer7 targeted to the mitochondrial matrix was treated or not with H_2_O_2,_ and oxidation of the reporter was estimated as described in Figure 1. The gradients of extracellular-to-cytosol (300:1) and cytosol-to-matrix (5:1) upon addition of H_2_O_2_ to the growing media are indicated. (**b**) Strain PN513 (WT) transformed with plasmids p730 and p790 expressing MTS-HyPer7 and MTS-Ctt1, respectively, in the mitochondrial matrix was treated and analyzed as in Figure 1. For each strain, average data from three biological replicates are shown, with error bars (S.D.) displayed in Figure S3.

**Figure 4:**
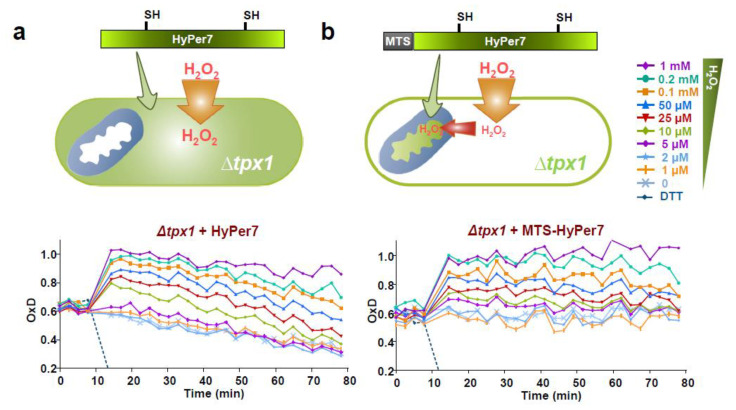
The levels of basal oxidation, OxD_0_, of HyPer7 and MTS-Hyper7 are very similar in cells lacking Tpx1. Strain SG5 (∆*tpx1*) transformed with p728 (**a**) or p730 (**b**) encoding HyPer7 or MTS-HyPer7, respectively, was grown and analyzed as in Figure 1. Average data from three biological replicates are shown, with error bars (S.D.) displayed in Figure S4.

**Figure 5:**
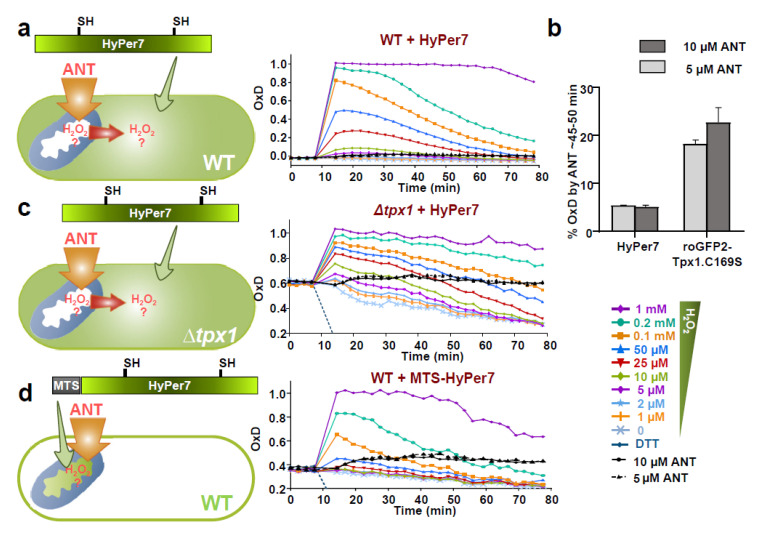
Detection of H_2_O_2_ produced at the mitochondria upon ANT treatment can be detected by MTS-HyPer7 but not by cytosolic HyPer7 unless the H_2_O_2_ scavenger Tpx1 is absent. (**a**) The indicated concentrations of H_2_O_2_ or ANT were directly added to MM cultures of strain HM123 + p728 (WT + HyPer7), and oxidation of the reporter was estimated as described in Figure 1; oxidations upon ANT treatments are represented with solid (10 μM ANT) or dashed (5 μM ANT) black lines. Average data from three biological replicates are shown, with error bars (S.D.) displayed in Figure S5. (**b**) Percentages of oxidation of the reporters HyPer7 and roGFP2-Tpx1.C169S upon ANT treatment were determined as described in Figure 1d between 45 and 50 min, from experiments of Figure 5a (HyPer7) and Figure S5d (roGFP2-Tpx1.C169S). (**c**,**d**) Cultures of strains SG5 + p728 (∆*tpx1*+ HyPer7), (**c**) and HM123 + p730 (WT + MTS-HyPer7) (**d**) were treated and analyzed as in (**a**).

HyPer has two excitation maxima at 420 and 500 nm and one emission peak at 516 nm [29], while HyPer7 has two excitation maxima at 400 and 499 nm and one emission peak at 516 [32]. Similarly, roGFP2 exhibits two excitation maxima at 400 nm and 475–490 nm when fluorescence emission is monitored at 510 nm. Either HyPer or roGFP2 oxidation was monitored using a fluorescence plate reader FLUOstar OMEGA (BMG Labtech). In both cases, we used excitation filters of 400–10 and 485BP12, combined with an emission filter of EM520. The experiments were performed as previously described [26]. Briefly, 190 µL of cultures at an OD_600_ of 1 were transferred to a 96-well imagine plate (Krystal Microplate™ 215003, Porvair Sciences) in as many wells as treatments were to be tested. The two excitation wavelengths were recorded, and after 4 cycles of approximately 2 min each, 10 µL of the corresponding treatments were added to accomplish the final concentrations of reagents indicated in Figure 1, Figure 2, Figure 3, Figure 4 and Figure 5. For the antimycin (ANT) treatment, a stock at 10 mM was prepared in DMSO, and it was freshly diluted in H_2_O to the final concentration for each experiment. In two wells, final concentrations of 50 mM dithiothreitol (DTT) and 1 mM of H_2_O_2_ were added as controls of fully reduced (red) and oxidized (ox) (see equation below). For each strain, we grew cultures of the wild-type background 972 and performed the same treatments on the 96-well plates; after recording, we subtracted the fluorescence values of the empty strain to those of the strain expressing the reporter. We determined the degree of sensor oxidation (OxD) as described in the following Equation (1):

(1) $$\mathrm{OxD}=\frac{\left(I_{sample\;488}\times I_{red\;405}\right)-(I_{sample\;405}\times I_{red\;488})}{(I_{sample\;405}\times I_{ox\;488})-(I_{sample\;405}\times I_{red\;488})-\left(I_{sample\;488}\times I_{ox\;405}\right)+\left(I_{sample\;488\;}\times I_{red\;405}\right)}$$

where ‘I_sample_’ represents the fluorescence intensity at 510 nm after excitation at either 405 nm or 488 nm of the treated sample (with H_2_O_2_ or ANT) at each time point, while ‘I_ox_’ and ‘I_red_’ represent the intensities of the control samples (with 1 mM H_2_O_2_ or 50 mM DTT) after 10 min of addition. In the case of *Δtrx1*, H_2_O was used as reduced control since the reaction of DTT in this cysteine-auxotrophic strain was not straightforward (data not shown). The bottom panels of Figure 1a–c represents the average OxD of each treatment between 10 and 15 min. When indicated (Figure 1d and Figure 5b), the OxD values of the cultures were expressed in a 0-to-100% scale, 0 being the OxD for the control treatment (0, H_2_O) for each reporter at the compared time point and 100% being the maximum level of oxidation upon 1 mM H_2_O_2_ at 10 min after stress imposition. All experiments were performed in biological triplicates using cells obtained from three independent cell cultures, except for Figure S5d,e, which were duplicates. For each new strain to be tested, HM123 carrying plasmid p728 or p730 was always added and analyzed in the same 96-well plate to use it as an internal control of basal probe oxidation.

### 2.5. Statistics

For in vivo fluorescence probe oxidation quantification, OxD, one independent culture of the strain of interest, was grown for each replicate. In all figure panels, values of mean of n = 3 or n = 2 (Figure S5d,e), are shown. For the statistical analysis, an unpaired *t*-test was performed. In supplementary figures, error bars for all the experiments (standard deviation, S.D.) are represented.

## 3. Results

### 3.1. Comparing the Performance of Different H_2_O_2_ Biosensors in the Same Model System—Expression of HyPer7 in Fission Yeast 

To quantitatively monitor the sensitivity of HyPer7 to H_2_O_2_ fluctuations, we expressed it in the cytosol of *S. pombe* cells under the control of the constitutive *sty1* promoter, as previously described for the original HyPer and for roGFP2-Tpx1.C169S. Moreover, we monitored its oxidation directly in exponentially growing cultures before and after the addition of extracellular peroxides, ranging from 1 to 1000 µM, using a fluorescence plate reader as previously described [26]. As shown in Figure 1 and Figure S1, the lower concentrations of extracellular H_2_O_2_ triggering statistically significant oxidation of HyPer, HyPer7, or roGFP2-Tpx1.C169S are 100, 5–10, and 1–2 µM, respectively. Therefore, even though HyPer7 is significantly more sensitive than the original HyPer, it does not reach the sensitivity of the peroxiredoxin-based biosensor. Nevertheless, HyPer7 is probably better suited to measure fluctuating waves of peroxides since its reduction is clearly faster than that of roGFP2-Tpx1.C169S.

### 3.2. HyPer7 Is Reduced by the Thioredoxin System

As shown in Figure 1, HyPer7 is actively recycled by *S. pombe*’s reducing systems, both before and after oxidation by peroxides. The thioredoxin and the glutaredoxin/glutathione systems maintain thiols in their reduced form in all cell types. To test which of both systems recycles HyPer7 in fission yeast, we expressed the reporter in the cytosol of cells lacking the main cytosolic glutaredoxin (Grx1) and the only glutathione reductase (Pgr1) (Figure 2a and Figure S2), or in cells lacking the cytosolic thioredoxins (Trx1 and Trx3) or the only thioredoxin reductase (Trr1) (Figure 2b). While the absence of Grx1 or Pgr1 has no effect on the basal levels of HyPer7 and do not slow down its reduction after H_2_O_2_ stress, the individual deletion of *trx1* has a long-term impact on the recycling of HyPer7 after stress imposition; as expected, the secondary thioredoxin Trx3 does not have an impact on HyPer7 reduction (Figure 2b). The lack of Trr1 also has a large impact on the reduction kinetics of the probe. We conclude that the thioredoxin system recycles HyPer7 in living cells (Figure 2c); on the contrary, the glutaredoxin-glutathione system recycles roGFP-Tpx1.C169S (Figure S2c), as previously described [26].

Regarding scavenging of cytosolic H_2_O_2_, we and others have previously demonstrated that catalase (Ctt1 in fission yeast) is required to detoxify high concentrations of applied peroxides, specifically those with an extracellular concentration higher than 0.1 mM. As shown in Figure 2d, when we compare the oxidation of HyPer7 in cells lacking Ctt1 (Figure 2d, ∆*ctt1* + HyPer7) and in wild-type cells (Figure 2a, WT + HyPer7), the sensor remains oxidized for longer times at extracellular concentrations ranging from 0.1 to 1 mM, while there are no significant differences at lower concentrations.

### 3.3. Expression of HyPer7 in the Mitochondria—Steady-State Levels of H_2_O_2_ in the Matrix

In order to determine the steady-state levels of H_2_O_2_ in the mitochondrial matrix, and also to study the fluxes of peroxides from the cytosol to the matrix and vice versa, we expressed HyPer7 at the mitochondrial matrix by fusing it to the mitochondrial targeting sequence (MTS) of Aco1, including the amino-terminal 60 amino acids of the matrix-resident protein aconitase 1 [35] (Figure 3a). Upon application of extracellular peroxides, the mitochondrial-targeted biosensor is capable of detecting oxidation, although ~5-times higher doses have to be applied to reach the oxidation levels of the cytosolic probe (compare WT + HyPer7 in Figure 2a with WT + MTS-HyPer7 of Figure 3a). This indicates that while there is a gradient of peroxides of 300:1 between the extracellular milieu and the cytosol, the gradient between the extracellular media and the mitochondrial matrix is 1500:1. Probe recycling after stress imposition is very similar to cytosolic HyPer7, suggesting that the matrix can efficiently reduce HyPer7 (compare Figure 3a with Figure 2a). 

Most importantly, the basal level of oxidation of MTS-HyPer7, OxD_0_, is also significantly altered, with ~35–40% of the probe being oxidized in a wild-type background (Figure 3a and Figure S3), while cytosolic HyPer7 is fully reduced during unperturbed conditions (see Figure 2a, WT + HyPer7). To rule out the lack of efficient recycling activities for oxidized MTS-HyPer7 in the matrix, we over-expressed catalase in this mitochondrial compartment by fusing it again to the MTS of Aco1. As shown in Figure 3b, the starting oxidation level of MTS-HyPer7 drops down to 0 when Ctt1 is also co-expressed in the matrix, strongly suggesting that the concentration of H_2_O_2_ in this site of production is significantly higher than in the cytosol. The extent of basal MTS-HyPer7 oxidation, ~35–40% OxD_0_, can be used as a non-quantitative readout of steady-state levels of H_2_O_2_, using cytosolic HyPer7 as a control. In wild-type cells, 50 μM of extracellular peroxides, which according to the 300:1 gradient correspond to ~0.15 μM of intracellular H_2_O_2_, are able to oxidize the probe up to ~40% (Figure 2a, WT + HyPer7). Therefore, we propose that the steady-state concentration of peroxides in the mitochondrial matrix is in the order of ~0.15 μM.

### 3.4. Role of the Peroxiredoxin Tpx1 in the Generation of Mitochondria-To-Cytosol H_2_O_2_ Gradients

Using biochemical approaches [25], as well as the basal oxidation levels of cytosolic roGFP2-Tpx1.C169S as a readout of the steady–state levels of H_2_O_2_ [26], we demonstrated that cells lacking Tpx1 display toxic concentrations of peroxides in the cytosol in the range of 0.2–0.3 µM. We expressed HyPer7 and MTS-HyPer7 in ∆*tpx1* cells (Figure 4 and Figure S4). The OxD_0_ for both reporters, cytosolic and mitochondrial, are very similar, ~60% oxidation in both compartments, suggestive of the presence of toxic concentrations of H_2_O_2_ irrespective of the presence of two membranes between the main site of peroxide production, the matrix, and the cytosol. This suggests that the different H_2_O_2_ levels in the matrix and the cytosol observed in wild-type cells (compare the OxD_0_ of HyPer7 with that of MTS-HyPer7 in wild-type strain; Figure 2a vs. Figure 3a) is caused by the efficient scavenging at the cytosol by the peroxiredoxin Tpx1, and the continuous production of reactive oxygen species at the mitochondria.

### 3.5. H_2_O_2_ Mitochondrial Bursts Can Be Detected by Mitochondrial MTS-HyPer7, or by HyPer7 in the Cytosol of Cells Lacking Tpx1

Since the ETC is an important source of H_2_O_2_ production, and its inhibitors can exacerbate peroxide production, we tested whether cytosolic HyPer7 is able to sense the H_2_O_2_ produced at and diffused out of the mitochondria. We treated cells with antimycin A (ANT), an inhibitor of CIII, and monitored HyPer7 oxidation in wild-type and ∆*tpx1* cells. Contrary to roGFP2-Tpx1.C169S, which is ~five-times more sensitive than HyPer7 to peroxides (see Figure 1) [26], HyPer7 can barely sense the leakage of H_2_O_2_ from the mitochondria upon addition of the ETC inhibitor ANT in wild-type cells (black lines in Figure 5a and Figure S5). Thus, while ANT treatments cause ~20% oxidation of cytosolic roGFP2-Tpx1.C169S (Figure S5d,e and Figure 5b), less than 5% of cytosolic HyPer7 is oxidized with the same treatments (Figure 5b). On the contrary, cytosolic HyPer7 is clearly oxidized upon ANT treatment in cells lacking the H_2_O_2_ scavenger Tpx1 (black lines in Figure 5c), confirming that H_2_O_2_ produced at the mitochondria is rapidly dispersed to the cytosol and degraded by Tpx1 in wild-type cells. 

MTS-HyPer7 expressed in wild-type cells, however, can monitor mitochondrial H_2_O_2_ production: ANT addition causes clear oxidation of this biosensor with very similar kinetics to the oxidation of cytosolic HyPer7 in ∆*tpx1* cells (compare black lines in Figure 5c,d).

## 4. Discussion

Redox biology is in need of reliable methods to measure in vivo fluctuations of H_2_O_2_. It may be quite easy to quantify large increases, which are normally linked to transient or permanent toxicity. Here, we test and compare two families of peroxide sensors expressed at similar levels in the same model system. By doing so, we demonstrate the advantages of one or the other and reach important conclusions regarding redox biology.

Thus, the peroxiredoxin-based sensor roGFP2-Tpx1.C169S is ~five times more sensitive than HyPer7 in monitoring increases in intracellular peroxides after applying H_2_O_2_ to the growth media, so that roGFP2-Tpx1.C169S oxidation can be detected upon 1–2 µM of H_2_O_2_ (Figure 1). The peroxiredoxin-based probe is partially oxidized in the cytosol of wild-type cells, in part due to the recycling of oxidized roGFP2, which depends on the glutaredoxin-glutathione system, but also due to the high sensitivity of peroxides to this probe [26]; HyPer7, on the contrary, is fully reduced during unperturbed conditions. Furthermore, the higher sensitivity of roGFP2-Tpx1.C169S explains why this peroxiredoxin-based probe can unambiguously detect in the cytosol the H_2_O_2_ produced at the mitochondria after ANT addition [26], but HyPer7 can barely suffer some minor changes (Figure 5b).

**Figure 6:**
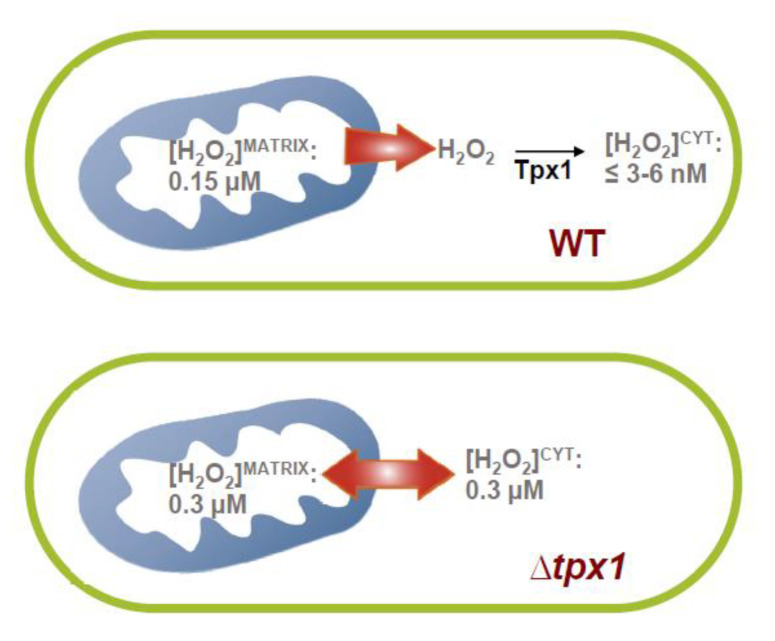
Scheme depicting the main finding of the use of the roGFP2-Tpx1.C169S and HyPer7 reporters in the quantification of basal and induced H_2_O_2_ levels and inter-compartmental H_2_O_2_ gradients. See Discussion for details.

The gradient of extracellular-to-intracellular peroxide is, on average, 300:1 [25]. Taking advantage of this knowledge, we can establish that roGFP2-Tpx1.C169S is able to detect intracellular H_2_O_2_ fluctuations in the low nanomolar (~3–6 nM) range, which is below the level where antioxidant redox signaling and toxicity occur (at least 200–300 nM of intracellular H_2_O_2_ is required to trigger activation of the pre-toxic Pap1 signaling cascade [25]). Since our probe is based on the natural H_2_O_2_ *S. pombe* scavenger Tpx1 [21], we propose that the capacity of this reporter to sense peroxides coincides with the steady-state levels of peroxides in the cytosol of wild-type cells, which are probably below and very close to 3–6 nM (Figure 6).

HyPer7 is, however, a very sensitive reporter, still detecting peroxide fluxes below the concentrations ruling signaling and toxicity events. Furthermore, it is rapidly recycled by the thioredoxin system so that mutants lacking cytosolic thioredoxins show slower reduction kinetics after stress imposition (Figure 2). A similar profile can be detected in cells lacking catalase at high concentrations of peroxides since this enzyme scavenges H_2_O_2_ ranging from an extracellular concentration of 0.1–1 mM (Figure 2d). In cells lacking Tpx1, for which we predicted an intracellular peroxide concentration of ~0.3 µM [25,26], HyPer7 is partially oxidized to ~60% (Figure 4a).

A remarkable advantage of the use of HyPer7 is that it can be efficiently imported to and recycled inside the mitochondrial matrix (Figure 3a). By doing so, we reach important conclusions about physiological peroxide fluxes and redox biology: (i) when extracellular H_2_O_2_ is applied, there is an additional peroxide gradient of 5:1 from the cytosol to the matrix, or of 1500:1 from the extracellular milieu to the matrix; (ii) MTS-HyPer7 is ~35–40% oxidized in the matrix of wild-type cells, which is ~half of the basal oxidation of cytosolic HyPer7 in ∆*tpx1* cells, with an OxD_0_ of ~0.6, or the levels of HyPer7 oxidation accomplished in wild-type cells upon 50 µM extracellular H_2_O_2_. We propose that the steady-state levels of peroxides in the matrix are ~0.15 µM. Thus, the main site of H_2_O_2_ production displays ~25–50 times higher levels of peroxides than the cytosol (Figure 6). 

Is this gradient caused by active H_2_O_2_ scavenging in the cytosolic compartment or by the limited permeability of the mitochondrial membranes? We propose that the gradient is caused by the former since HyPer7 and MTS-HyPer7 display similar OxD_0_ in cells lacking the peroxiredoxin Tpx1 (Figure 4 and Figure 6). This result suggests that mitochondrial H_2_O_2_ can diffuse out of the mitochondria, but it is rapidly scavenged by Tpx1 at the cytosol. In fact, this diffusion is rapid, as shown with ANT treatments, and only endogenous Tpx1 or the roGFP2-Tpx1.C169S probe contain a sensitive thiol group (thiol switch) in their cysteine 48 as to detect waves of physiological peroxides [26] (Figure 6). This is an important concept in redox signaling, which reinforces the idea that only a handful of proteins such as peroxiredoxins can sense peroxide waves emanating from the mitochondria and transduce the signal to control cell signaling cascades.

## 5. Conclusions

We have compared the H_2_O_2_ reporters HyPer7 and roGFP2-Tpx1.C160S in *S. pombe.* Most results are outlined in Figure 3a and Figure 6. Thus, the peroxide gradient from the extracellular media to the matrix, 1500:1, is five times higher than that from the extracellular media to the cytosol. Thanks to the use of these reporters, we speculate that the steady-state H_2_O_2_ level in the matrix is around 0.15 µM, while the concentration in the cytosol is in the low nanomolar range; this gradient is caused by the scavenging activity of the cytosolic peroxiredoxin, Tpx1. Thus, in cells lacking this scavenger, the mitochondria-to-cytosol gradient disappears, with matrix and cytosolic H_2_O_2_ concentrations being in the order of 0.3 µM. The future use of these reporters in other subcellular compartments (intermembrane space or endoplasmic reticulum) and in different model systems (such as mammalian cells) will be required to generalize all the findings of our work.

## Data Availability

The data presented in this study are available on request to the corresponding author.

## Supplementary Materials

The following are available online at https://www.mdpi.com/article/10.3390/antiox10050731/s1, Figures S1–S5—graphs of main figures with error bars; Table S1—Strains used in this study.
