# Supplementary material for: The Mitochondria-to-Cytosol H2O2 Gradient Is Caused by Peroxiredoxin-Dependent Cytosolic Scavenging
Source: Antioxidants (Basel). 2021 May 6;10(5):731. doi: 10.3390/antiox10050731 (PMC8148214; doi:10.3390/antiox10050731)
Supplement: Supplementary file 1 [file antioxidants-10-00731-s001.zip › antioxidants-1179592-supplementary.pdf]

SUPPLEMENTARY DATA

The mitochondria-to-cytosol H2O2 gradient is caused by peroxiredoxin-dependent cytosolic scavenging

de Cubas et al.

It includes:

5 supplementary Figures

1 supplementary Table

Supplementary References

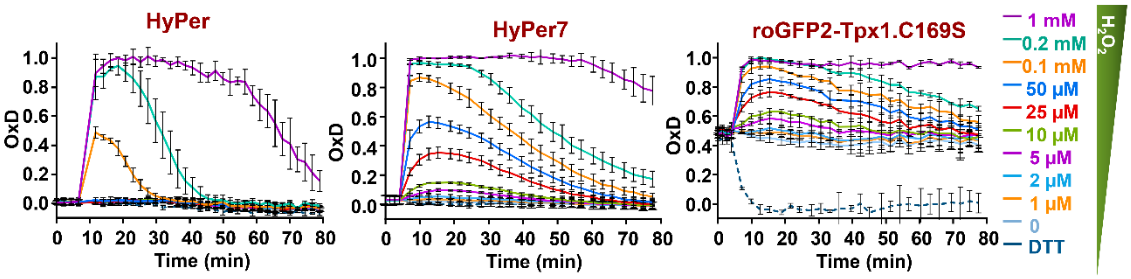

Figure S1. Expression of HyPer, HyPer7 and roGFP2-Tpx1.C169S in fission yeast. Wild-type strain HM123 was transformed with plasmids p605, p728 and p407.C169S to express HyPer, HyPer7 and roGFP2-Tpx1.C169S, respectively. Oxidation of the reporter was estimated as described in Figure 1. Data from three biological replicates with error bars (S.D.) are shown.

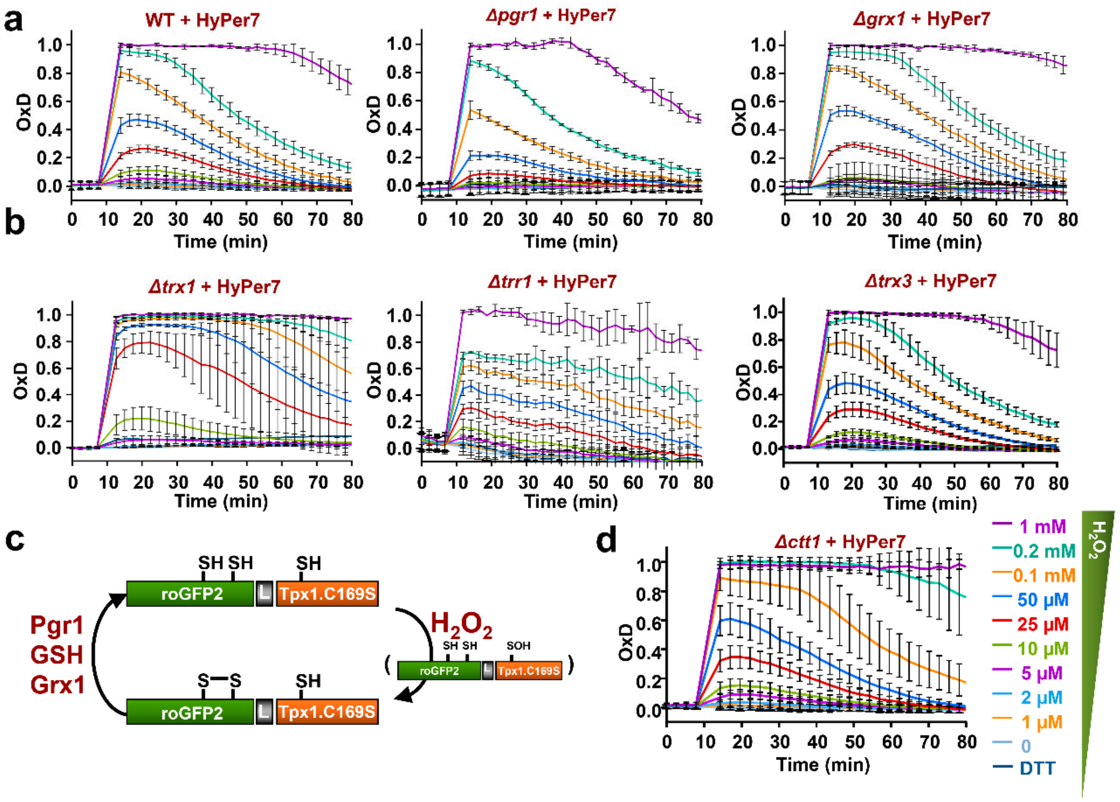

Figure S2 (**a, b**) Strains HM123 (WT), AD88 (Δ*pgr1*), SG154 (Δ*trx1*), SB469 (Δ*grx1*), SG152 (Δ*trr1*), and MC184 (Δ*trx3*), transformed with plasmid p728 expressing cytosolic HyPer7, were treated or not with H_2O_2 and oxidation of the reporters was estimated as described in Figure 1. Data from three biological replicates with error bars (S.D.) are shown. (**c**) Scheme depicting the role of the glutathione system as the electron donor in roGFP2-Tpx1.C169S recycling. (**d**) Strain EP160 (Δ*ctt1*) transformed with plasmid p728 expressing cytosolic HyPer7 was treated or not with H_2O_2 and oxidation of the reporter was estimated as described in Figure 1. Data from three biological replicates with error bars (S.D.) are shown.

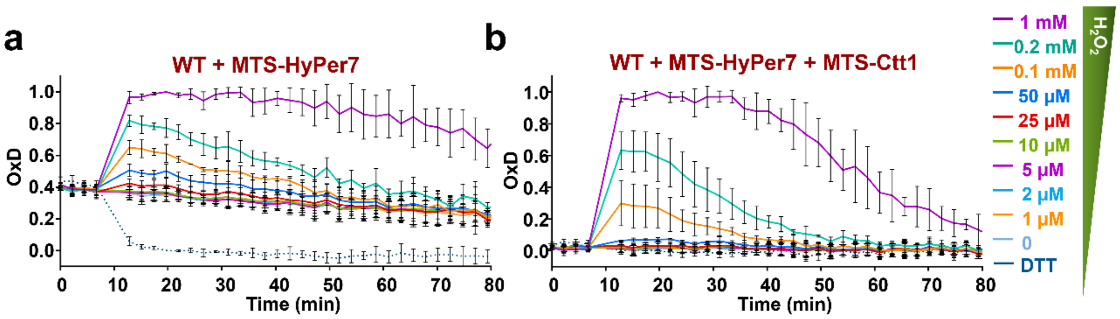

Figure S3. (**a**) Strain HM123 (WT) transformed with plasmid p730 expressing MTS HyPer7 targeted to the mitochondrial matrix was treated or not with H_2O_2 and oxidation of the reporter was estimated as described in Figure 1. (**b**) Strain PN513 (WT) transformed with plasmids p730 and p790 expressing MTS-HyPer7 and MTS-Ctt1, respectively, in the mitochondrial matrix was treated and analyzed as in Figure 1. Data from three biological replicates with error bars (S.D.) are shown.

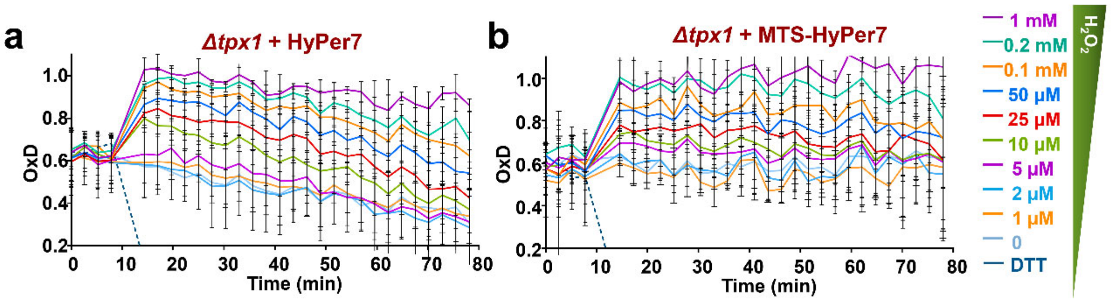

Figure S4. The levels of basal oxidation, OxD_0, of HyPer7 and MTS-Hyper7 are very similar in cells lacking Tpx1. Strain SG5 (Δ*tpx1*) transformed with p728 (**a**) or p730 (**b**) encoding HyPer7 or MTS-HyPer7, respectively, was grown and analyzed as in Figure 1. Data from three biological replicates with error bars (S.D.) are shown.

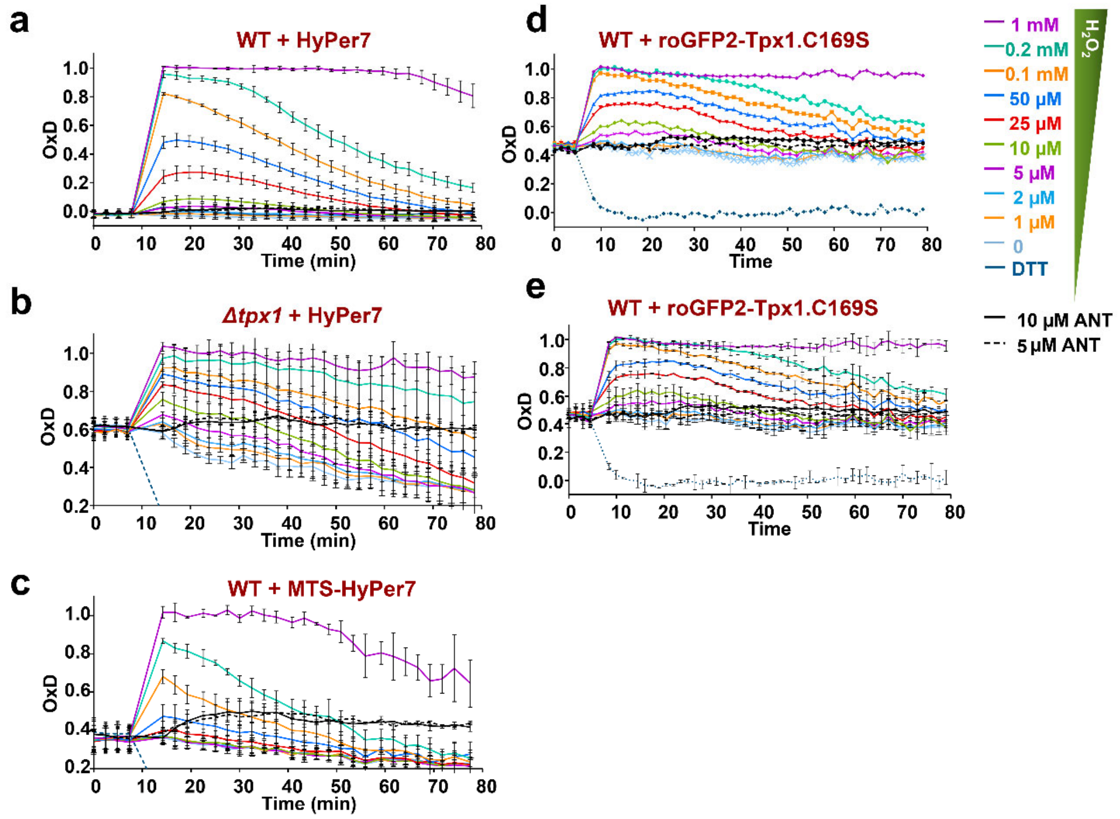

Figure S5. The indicated concentrations of H_2O_2 or ANT were directly added to MM cultures of strains HM123 + p728 (WT + HyPer7) (**a**), SG5 + p728 (Δ*tpx1*+ HyPer7) (**b**), and HM123 + p730 (WT + MTS-HyPer7) (**c**), and HM123 + p407.C169S (WT + roGFP2-Tpx1.C169S) (**d** ,**e**) and oxidation of the reporters was estimated as described in Figure 1; oxidations upon ANT treatments are represented with solid (10 μM ANT) or dashed (5 μM ANT) black lines. Data from three (a, b, c,) or two (d, e) biological replicates with error bars (S.D.) are shown.

Table S1. Strains used in this study

Strain	Genotype	Origin
972	*h-*	[1]
HM123	*h- leu1-32*	Lab Stock
AD88	*h+ pgr1::natMX6 leu1-32*	[2]
SG154	*h- trx1::kanMX6 leu1-32*	This work
SB469	*h- grx1::kanMX6 leu1-32*	This work
MC184	*h+ trx3::natMX6 leu1-32*	This work
SG152	*h+ trr1::ura4 ura4-D18 leu1-32*	This work
EP160	*h- ctt1::ura4 ura4-D18 leu1-32*	[2]
PN513	*h- ura4-D18 leu1-32*	Paul Nurse lab
SG5	*h+ tpx1::natMX6 leu1-32*	[3]
MJ2	*h- trx1::kanMX6 ura4-D18 leu1-32*	[4]
JA366	*h+ leu1-32*	Lab Stock
IC38	*h- grx1::kanMX6*	[3]
NG24	*h- caf4+::ura4+ ura4-D18*	[5]
666	*h+ ura4D-18 leu1-32 ade6-M210*	Bioneer colection
SG265	*h+ trx1::kanMX6, trx3::natMX6*	This work
SGS257	*h- trx3::natMX6*	[4]
MJ15	*h+ trx1::kanMX6*	[4]

SUPPLEMENTARY REFERENCES

1. Leupold, U. Genetical methods for *Schizosaccharomyces pombe*. *Methods Cell Physiol.* **1970**, *4*, 169-177.
2. Carmona, M.; de Cubas, L.; Bautista, E.; Moral-Blanch, M.; Medrano-Fernandez, I.; Sitia, R.; Boronat, S.; Ayte, J.; Hidalgo, E. Monitoring cytosolic H2O2 fluctuations arising from altered plasma membrane gradients or from mitochondrial activity. *Nat Commun* **2019**, *10*, 4526, doi:10.1038/s41467-019-12475-0.
3. Calvo, I.A.; Boronat, S.; Domenech, A.; Garcia-Santamarina, S.; Ayte, J.; Hidalgo, E. Dissection of a redox relay: H2O2-dependent activation of the transcription factor Pap1 through the peroxidatic Tpx1-thioredoxin cycle. *Cell Rep* **2013**, *5*, 1413-1424, doi:10.1016/j.celrep.2013.11.027.
4. Garcia-Santamarina, S.; Boronat, S.; Calvo, I.A.; Rodriguez-Gabriel, M.; Ayte, J.; Molina, H.; Hidalgo, E. Is oxidized thioredoxin a major trigger for cysteine oxidation? Clues from a redox proteomics approach. *Antioxid Redox Signal* **2013**, *18*, 1549-1556, doi:10.1089/ars.2012.5037.
5. Calvo, I.A.; Gabrielli, N.; Iglesias-Baena, I.; Garcia-Santamarina, S.; Hoe, K.L.; Kim, D.U.; Sanso, M.; Zuin, A.; Perez, P.; Ayte, J., et al. Genome-wide screen of genes required for caffeine tolerance in fission yeast. *PLoS One* **2009**, *4*, e6619.
